# Exploring Change in Trend of Homicide Incidence Rate in Iran from 2006 to 2016: Applying Segmented Regression Model

**DOI:** 10.34172/jrhs.2020.12

**Published:** 2020-05-05

**Authors:** Hajar Nazari Kangavari, Abdolrazagh Barzegar, Seyed Davood Mirtorabi, Mohammad Reza Ghadirzadeh, Mehdi Forouzesh, Niloufar Taherpour, Fatemeh Shahbazi, Seyed Saeed Hashemi Nazari

**Affiliations:** ^1^Department of Epidemiology, School of Public Health, Iran University of Medical Sciences, Tehran, Iran; ^2^Legal Medicine Research Center, Legal Medicine Organization, Tehran, Iran; ^3^Department of Addiction Studies, School of Advanced Technologies in Medicine, Tehran University of Medical Sciences, Tehran, Iran; ^4^Department of Epidemiology, School of Public Health and Safety, Shahid Beheshti University of Medical Sciences, Tehran, Iran; ^5^Safety Promotion and Injury Prevention Research Center, Department of Epidemiology, School of Public Health and Safety, Shahid Beheshti University of Medical Sciences, Tehran, Iran

**Keywords:** Incidence rate, Homicide, Iran

## Abstract

**Background:** Murder is one of the public health problems. According to the WHO reports, murder is fourth leading cause of death among young people. The aim of this study was applying joint point regression model to study trend of homicide mortality in Iran, 2006-2016.

**Study design:** A cross-sectional panel (pseudo-panel) study.

**Methods:** Homicide data during 2006 to 2016 were extracted from Iranian legal medicine organization. Trends of homicide incidence were summarized by annual percent change (APC) and average annual percent change (AAPC) using non-linear segmented regression model.

**Results:** Totally, 26918 homicide cases occurred during the period from 2006 to 2016. The highest and lowest frequency was related to the 15-29 yr (46.5%) and 0-4 yr (1.5%) age groups, respectively. The homicide incidence rate of the country in 2016 was 2.81 per 100,000. The four provinces of Sistan & Baluchistan, Khuzestan, Kerman and Ilam had the highest incidence rate in 2016, respectively. During the study period, the incidence rate of homicide in Iran and men have been significantly decreased (APC: -2.8% (95% CI: -3.9, -1.7) and -3.2% (95% CI: - 4.5, -1.8) respectively (*P* <0.001)).

**Conclusion:** The pattern of homicide rate has a downward trend in the country. Moreover, the varying observed trends in some provinces can be due to the variability in mental, geographical, socio-economic and cultural conditions in each region.

## Introduction

Murder or homicide is the act of killing a person by another to cause death or serious harm to her/him ^1^. In other words, homicide means that someone is being killed illegally ^2^. According to the WHO report, about 470,000 homicides occur each year in the world ^3^. Every year about 200,000 murders occur among youth 10-29 yr which accounts for about 43% of all homicides that is the fourth leading cause of death for people in this age group. About 84% of homicides occur in males aged 10-29 years. Therefore, the incidence rate of homicide among men was more than women around the world. During 2000-2012 incidence rate of homicide among youth has decreased in most of the countries. However, this decrease is mostly related to high and middle-income countries ^4^. The highest incidence rate of homicide was in El Salvador, Honduras, Venezuela and Colombia, respectively ^5^.

Moreover, homicide in Iran occurred in 3630 people (4.1 per 100,000) in 2015 that it was more common in men (6.8 per 100,000) compared to women. While women more than 60 yr old had highest incidence rate of homicide (6.5 per 100,000) ^6^. In Iran in 2019, about 80.3% of the victims were men and the mean age of victims were 32.4 year. Besides, the most root cause of murder was quarrel and most of murders were carried out using a weapon like firearms (46%) and stab wounds (29%), respectively ^7^.

According to the report of variety studies in Iran and other countries regarding homicide different risk factors like gender, age, psychiatric disorders, climatic and ecological conditions, socio-economical level, cultural factors, poverty, individual violence and family breakdown are among the most mentioned risk factors of Homicide or murder ^8-12^. Moreover, among Europeans, annual alcohol consumption positively associated with the rate of mortality due to homicide ^13^. In Iran a study in Isfahan showed the living in rural area, living in crowding family, lack or low level of literacy and low-level income, history of violence and drugs in family were predisposing risk factors for homicide ^14^.

According to the past studies about homicide, the incidence rate of homicide has varied over the years and has been influenced by many economic, social and other factors ^15-19^. In Iran all of previous studies about homicide were cross-sectional that conducted during one year, the trend of incidence rate changes of homicide in the country has not been investigated so far ^14,20^.

According to the importance of homicide in the world especially in Iran, we were going to depict the epidemiology of homicide in Iran and investigate any changes in the homicide’s incidence rate trend by applying joint point regression during 2006-2016.

## Methods

The study was a cross-sectional panel (pseudo-panel) case. The homicide data were extracted from Iranian legal medicine organization from 2006 to 2016. According to the national laws in Iran on legal cases of death, all suspicious deaths should be referred to as the Legal Medicine Organization and death certificate is just issued by this organization. We extracted information of all available and registered homicide cases from Iranian legal medicine organization during the years 2006 to 2016 and the annual mortality rates were calculated. Moreover, to adjust for any differences in age distribution during the study period and also for the sake of comparison with other countries, directly standardized rates using the latest standard population of WHO were also measured ^21^.

First, the homicide data were checked for completeness and any inconsistencies. The entered data were cleaned and edited before subsequent analysis. Second, homicide rates during 2006 to 2016 were estimated by dividing the number of homicides by population.

We obtained census population counts for Iran for 2006, 2012 and 2017 and carried out a linear interpolation of the census population counts to obtain population denominators for the intervening years^22^. All rates were calculated per 100,000 populations. We subsequently, directly standardized overall rates by age to the world (WHO 2000-2015) standard population to enable comparability between years relevant to this analysis and also rates from other countries.

Third, we applied the Joinpoint regression model for determining changes in the trend of the homicide incidence rate during the study period. The number of change points were selected based on the permutation test with 5000 iterations. Trends of homicide incidence were summarized by annual percent change (APC) and average annual percent change (AAPC). Joinpoint (V.4.6) software was used for trend analysis at 0.05 level of significance. Jointpoint regression model was fitted to the data using maximum likelihood estimation and for choosing the best model (comparing linear and log-linear models) Bayesian Information Criterion (BIC) were used. Summary statistics such as frequency and percentage were computed for qualitative data and also mean and standard deviation were computed for quantitative data. Annual percent change in a log-transformed model measures the annual percentage change in rate. It is estimated according to the following formula:

Log (Rate[y]) = b0 + b1 *y where log(rate[y]) is the natural log of rate in year [y] and the APC from year [y] to year [y+1] is equal to: (e^ b1^-1)*100.

This study was registered at Legal Medicine Research Center in Iran and was approved by the Ethics Committee of the Legal Medicine Organization (IR.LMO.REC.IR.LMO. REC.1396.22).

## Results

Totally, 26918 homicide cases (81.1% in males and 18.9% in females) occurred during 2006 to 2016. The average age of the homicide cases during the study period was 32.88 **±**(0.09) and most of the victims were 15-29 and 30-49 year. During the study period the relative frequency of homicide in men was more than women.

**Figure 1:**
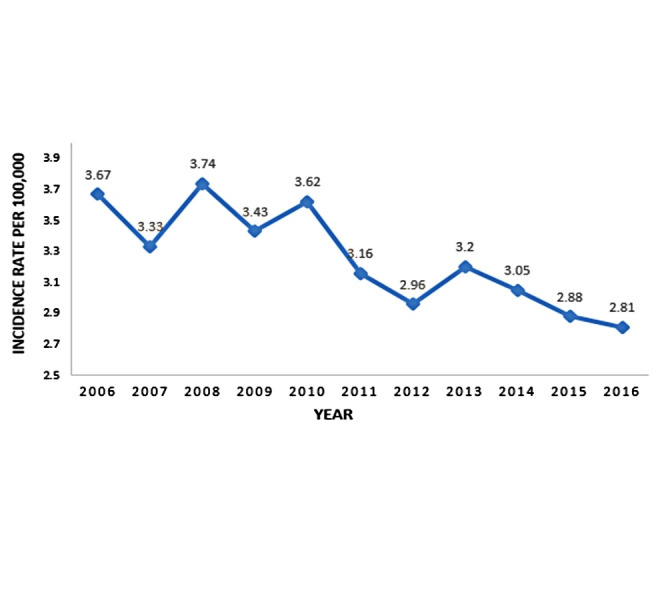
Incidence rate of Homicide in Iran from 2006 to 2016 (per 100,000)

The highest and lowest incidence rate of homicide was in 2008 and 2016 respectively (Figure 1). After analyzing homicide mortality rate by age groups, results showed that in 2016 highest and lowest occurrence were in 15-29 yr (4.37 per 100,000) and 0-4 yr (0.66 per 100,000) age-group, respectively. Age-group of 15-29 and 30-49 yr had the highest homicide mortality rates compared to other age-group (Table 1).

**Table 1:** Homicide mortality rate by age groups in Iran from 2006 to 2016 (per 100,000)

**Age-groups**	**0-4**		**5-14**		**15-29**		**30-49**		**50-69**		**≥70**	
**Year**	**Number**	**Rate**	**Number**	**Rate**	**Number**	**Rate**	**Number**	**Rate**	**Number**	**Rate**	**Number**	**Rate**
2006	33	0.67	63	0.52	1325	5.31	870	4.81	260	3.56	98	3.99
2007	24	0.43	68	0.57	1156	4.69	800	4.27	245	3.19	80	3.14
2008	40	0.69	52	0.44	1310	5.38	954	4.93	266	3.29	77	2.92
2009	31	0.52	51	0.44	1255	5.21	808	4.03	267	3.14	96	3.51
2010	33	0.54	63	0.55	1286	5.39	928	4.47	286	3.20	85	2.99
2011	28	0.45	42	0.37	1143	4.82	840	3.91	238	2.53	83	2.81
2012	37	0.58	45	0.39	1036	4.52	828	3.71	240	2.45	68	2.27
2013	32	0.49	57	0.49	1085	4.90	923	3.98	267	2.62	100	3.30
2014	43	0.64	62	0.53	1062	4.96	864	3.59	276	2.60	72	2.35
2015	34	0.49	49	0.41	985	4.75	873	3.49	272	2.46	63	2.02
2016	47	0.66	66	0.55	876	4.37	933	3.59	253	2.19	66	2.09

During 11 years, most of the homicides occurred using firearms and after that cold weapon like knife and sharp things had the second rank. The frequency of firearms was 45.2 % and knife and sharp things was 32.7% from 2006 to 2011 and from 2012 to 2016 frequency of homicide by firearms was 45.28% and knife and sharp things drop to 27.7%. The method with the lowest frequency was electrocution (0.03%).

After calculating the incidence rates by provinces of Iran in 2016, was observed that the four provinces of Sistan & Baluchistan, Khuzestan, Kerman and Ilam had the highest incidence rates in 2016, respectively (Table 2).

**Table 2:** Incidence rate of Homicide by Provinces of Iran in 2016 (per 100,000)

**Province**	**Number**	**Rate**
Chaharmahal and Bakhtiari	19	2.0
West Azarbaijan	126	3.86
Fars	155	3.2
Esfahan	84	1.64
Gilan	60	2.37
Qazvin	24	1.88
Ardabil	11	0.87
Sistan and Baluchestan	341	12.29
East Azarbaijan	60	1.53
Ilam	24	4.14
Kerman	154	4.36
Hormozgan	52	2.93
Hamadan	40	2.30
Golestan	39	2.09
Semnan	11	1.57
Kohgiluyeh and Boyer-Ahmad	19	2.66
South Khorasan	6	0.78
Markazi	59	4.13
Kermanshah	62	3.18
Zanjan	12	1.13
Bushehr	44	3.78
Khuzestan	218	4.63
Yazd	15	1.32
North khorasan	10	1.16
Razavi Khorasan	142	2.21
Alborz	49	1.81
Lorestan	66	3.75
Tehran	229	1.73
Mazandaran	43	1.31
Qom	19	1.47
Kordestan	40	2.50
Total	2244	2.81

After comparing the age-standardized incidence rate by gender, was observed that generally, homicide rates were significantly higher among men compared to women (4.17, 95% CI: 4.04, 4.3, incidence rate- ratio of men to female).

During the study period, joint regression analysis showed the incidence rate of homicide in Iran and men have been significantly decreased (APC: -2.8% (95% CI: -3.9, -1.7) and -3.2% (95% CI: - 4.5, -1.8) respectively (*P* <0.001). However, the incidence rate of homicide in women was almost constant and the trend in this subgroup was not statistically significant (*P* =0.100). In other words, the incidence rate of homicide 2.8% and 3.2% significantly decreased per year in the whole country and men, respectively but incidence rate of homicide in women was almost constant. (Table 3).

**Table 3:** Trend analysis of homicide mortality by gender and age strata from 2006 to 2016 in Iran

**Cohort**	**Segment**	**Time period**	**APC (95% CI)**	**P Value**	**AAPC (95% CI)**	**P value**
Total (Iran)	1	2006-2016	-2.8 (-3.9, -1.7)	0.001	-2.8 (-3.9, -1.7)	0.001
**Gender**						
Male	1	2006-2016	-3.2 (-4.5, -1.8)	0.001	-3.2 (-4.5, -1.8)	0.001
Female	1	2006-2016	-1.0 (-2.0, 0.10)	0.100	-1.0 (-2.0, 0.1)	0.100
**Age groups (yr)**						
0-4	1	2006-2016	0.7 (-2.9, 4.3)	0.700	0.7 (-2.9, 4.3)	0.700
5-14	1	2006-2016	-0.6 (-3.9, 2.9)	0.700	-0.6 (-3.9, 2.9)	0.700
15-29	1	2006-2016	-1.3 (-2.6, -0.1)	0.001	-1.3 (-2.6, -0.1)	0.001
30-49	1	2006-2014	-3.3 (-5.9, -0.6)	0.001	-2.9 (-7.1, 1.4)	0.200
	2	2014-2016	-1.3 (-23.2, 26.9)	0.900		
50-69	1	2006-2012	-0.5 (-8.5, -1.4)	0.001	-4.2 (-6.9, -1.4)	0.001
	2	2012-2016	-0.3 (-9.5, 4.1)	0.300		
≥70	1	2006-2016	-5.3 (-0.8, -2.6)	0.001	-5.3 (-8.0, -2.6)	0.001

**Figure 2:**
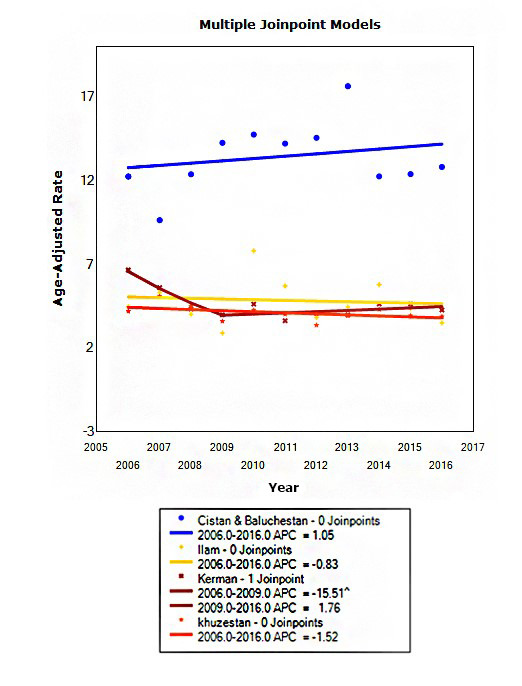
Trend of Homicide logarithmic changes in four provinces of Iran with high incidence rate from 2006 to 2016

We also estimated the trend of homicide in the four provinces with highest homicide incidence rates including Sistan and Baluchistan, Ilam, Kerman and Khuzestan. After analyzing the four mentioned provinces, a model with one joint point was chosen as the best model for Kerman province but for other provinces models without any break-point were chosen as the best model. In Kerman, the study period was divided into two periods from 2006 to 2009 and 2009 to 2016. The first slope from 2006 to 2009 revealed that incidence rate, about 16% decreased (*P* =0.030). In other four provinces, the slopes of trends were not statistically significant and incidence rates were constant during the study period (Figure 2).

According to analysis of homicide mortality trends by age strata (Table 3), zero joint point model were found to be the best fitted model in the age groups 15-29 and ≥70 yr, whereas changes in two age strata 0-4 and 5-14 yr were not statistically significant. In 30-49 and 50-69 yr, 1 joint point models were obtained as the best models. For age group, 30-49 yr, homicide mortality rates presented a significant decrease of about 3% per year from 2006 to 2014 and non-significant decline from 2014 onwards. Estimation by Joint point analysis in 50-69 yr determined as a significant decline of 0.5% per year from 2006 to 2012 but a non-significant decrease from 2012 onwards.

## Discussion

The current study showed trend of homicide incidence rate in Iran during 2006 to 2016. Mean age of homicide cases during 2006 to 2016 was 32.88 **±**(0.09) yr and most of victims were 15-29 and 30-49 yr old. Most of the homicide cases were occurred in 15-44 yr’ age group in East-Mediterranean ^23^. In addition, in Isfahan, most of victims were 15-29 yr old ^24^. In high-income countries most of homicide cases occurred in the age group of 25- 34 yr old and after that in 75 yr and more ^25^. In the most of countries in the world, most of homicide occurs among youth and middle-aged. The reasons for this are complex but economic and social pressures, drug and alcohol abuse, media violence access, weapon availability especially cold weapon, puberty age among teens and modern cultures which values material goods and personal success and leads to unhealthy competitions among youth and teens maybe affect occurrence of homicide among mentioned age group.

In this study, similar to other studies conducted in parts of Europe and Asia, homicide occurred much more among males (66% to 87%) ^26-30^. According to the WHO reports in 2016, about 83% of homicide occurred among males which their results are in agreement with the current study ^4^. The most frequent method of homicide was using firearms and this results agreed with another study that was conducted in United-States ^31^ while another studies in Iran reported that the most common homicide’s method was cold weapon like knife^24,9^. The reason for this difference is that current study has been done on the national level but previous studies have been conducted in the provincial levels.

According to the results of break-point analysis, generally, incidence rate of homicide in Iran and among men were without break-point and have decreased. Moreover, incidence rate of homicide in country has been significantly decreasing from 3.67 per 100,000 to 2.81 per 100,000 during 2006 to 2016 while in Russia the incidence rate of homicide in this country from 2001 to 2009 was fairly constant ^32^. In the United-States, homicide incidence rate among males and age group of 18-49 yr was more than others during 1980 to 2008. Generally, in that country trend of homicide incidence rate has been along with the ups and downs during 1980 to 2008. Therefore, in 1980 it has been increasing, and then peaked in 1991, after which the trend has been decreasing in 2008 ^15^. We can say that homicide trend changes in different countries depend on national factors such as serious socio-economic and political changes, unemployment, alcohol abuse especially in European countries, and tribal traditions, especially in Asian and African countries. Changes in these factors during the time induce some changes in occurrence of crimes such as homicide. About Iran, we can say existence of laws relevant to preventing different types of violence such as children maltreatment laws against child marriage or legal age of women or men’s marriage, youth violence laws against weapons on school permission or gang or criminal group membership, sexual violence laws against rape and victim’s laws about providing for victim compensation can affect the reduce of homicide in the country.

Results of annual percent changes (APC) analysis revealed that homicide incidence rate decreased about 2.8% and 3.2% in the whole of Iran and men, respectively from 2006 to 2016 while the result of Lotufo and Bensenor’s study in Brazil during 12 yr showed that among men 15-44 yr, APC increased 4.7% during 1996 to 2001 and decreased 14.6 % during 2001 to 2007 ^33^.

Furthermore, APC analysis by age groups in current study showed that homicide mortality rates among people in 30-49 yr in Iran have decreased about 3% per year from 2006 to 2014 and in 50-69 yr this rate have declined 0.5% per year from 2006 to 2012. This finding was in contrast to another study’s results in Puerto Rico. The result of APC analysis showed significant increase from 2007 onwards especially in the 20-24 and 25-29 yr’ age groups. Moreover, AAPC was showed significant increase from 2007 onward across 20 to 44 yr’ age group ^34^. According to the results of current study, incidence rate of homicide in Iran have been different by provinces so that Sistan and Baluchistan, Khuzestan, Kerman and Ilam provinces had the highest incidence rate in 2016, respectively. This finding is in line with other studies in which, homicide incidence rate in other countries was different at the provincial level. Besides, incidence rate of homicide in western regions of Norway was lower than the overall average in the country^31,35,36^.

The trend of homicide has been decreasing throughout the country but the increasing trend in some of border provinces was observed which seems to be the main challenge in controlling this phenomenon in these provinces which are in the neighborhood with countries who are at war or are involved with illegal drug and human trading. Another cause of high incidence in the four mentioned provinces of Iran can be tribal conflict, honor killing and high prevalence of consuming drugs and easy access to weapons in the border regions of the country ^37,38^.

Future studies are suggested to be carried out on the following cases:

1. Assessment of incidence rate by provinces to recognize hotspot regions and also spatial analysis

2. Assessment of risk factors related to occurrence of murder in the form of analytical studies for intervention and policy making

Use of the most complete, accurate data of homicide in the country without sampling process is the strength of the study and lack of access to other risk factors of homicide occurrence such drug and alcohol abuse, income status, employment and unemployment status are the weaknesses of the study.

## Conclusion

The incidence rate of homicide in Iran has been decreasing but currently, the incidence of homicide in some border provinces is high compared to the overall average in the country. Although, the decline in incidence rate of homicide over the past decade in the country has been good but not enough. Authorities should apply different policies for controlling this issue, like recognition of homicide’s hotspot regions and controlling the risk factors that lead to the occurrence of murder such as attention to social and economic issues, violence status, drug addiction and easy access to it, psychiatric disorders, poverty, unemployment, social inequality and false tribal traditions especially in provinces with higher than average rate of homicide and planning for strategies to prevent youth violence such as, counseling, vocational training, family therapy, training health care workers to identify and refer youths at high risk for violence and activities and polices to mitigate the effect of rapid social change and tackle gun violence among youth.

## Acknowledgements

The authors would like to thank the Iranian Legal Medicine Research Center and Iranian Legal Medicine Organization which participated in this project and supported this study and provided the necessary data.

## Conflict of interest

None declared.

## Funding

This study is financially supported by the Research Center of Legal Medicine Organization of Iran.

## Highlights

- The incidence trend of homicide in Iran decreasing from 3.67 to 2.81 per 100,000 from 2006 to 2016.
- The four provinces of Sistan & Baluchistan, Khuzestan, Kerman and Ilam had the highest incidence rate in 2016, respectively.
- Homicide rates were significantly higher among men compare to women (4.17, 95% CI: 4.04 to 4.3, incidence rate- ratio)
- Joint regression analysis showed, incidence rate of homicide 2.8% and 3.2% significantly decreased per year in Iran and men, respectively.
